# Poster Session II A333 PATTERNS AND DIAGNOSTIC YIELD OF ESOPHAGEAL MOTILITY DISORDERS: A RETROSPECTIVE HIGH-RESOLUTION MOTILITY STUDY

**DOI:** 10.1093/jcag/gwaf042.333

**Published:** 2026-02-13

**Authors:** S Alobaid, Y Alotaibi, Y Han, A Alhazmi, A A Saqah, L Alrabghi, M Ismail, O Pacyna, N Anwar, R Elzaanoun, M R Jouid, K McIntosh, R Mortuza, R Sedano

**Affiliations:** Western University, London, ON, Canada; Western University, London, ON, Canada; Western University, London, ON, Canada; Western University, London, ON, Canada; Western University, London, ON, Canada; Western University, London, ON, Canada; Western University, London, ON, Canada; Western University, London, ON, Canada; Western University, London, ON, Canada; Western University, London, ON, Canada; Western University, London, ON, Canada; Western University, London, ON, Canada; Western University, London, ON, Canada; Western University, London, ON, Canada

## Abstract

**Background:**

Esophageal motility disorders are underreported in Canada and remain a clinical challenge that impacts quality of life. High-resolution esophageal manometry (HREM) is the gold standard for their diagnosis.

**Aims:**

Primary Aim: To determine the prevalence of esophageal motility disorders in a population of patients from Southwestern Ontario referred for (HREM).

Secondary Aim: To evaluate various HREM parameters across normal and abnormal studies.

**Methods:**

A retrospective analysis was conducted on patients who underwent HREM at St. Joseph Hospital, in London, Ontario, from Jan 1^st^, 2014, until Sep 15, 2024. Data was extracted from a motility database of patients who had undergone HREM. The data included patient characteristics, medications, symptoms, and comorbidities, along with indications for HREM, HREM parameters, measurements, and the final diagnosis.

**Results:**

A total of 1352 patients were included in the final analysis, with the majority being Female (822/1352, 60.8%). Mean age was 54.12 (18-96) years, with an average weight of 80.4 kg (25.4 - 185 kg) and a mean height of 168.03 cm (81–201 cm).

The most common indication for HREM was dysphagia (587/1352;43.4%), followed by typical GERD symptoms, both heartburn and regurgitation (514/1352;38%).

HREM revealed abnormal findings in 582/1352 (43%) of patients, whereas 770/1352 (57%) had normal study results. Ineffective esophageal motility (IEM) was the most prevalent diagnosis (98/1352; 7.2%), followed by achalasia (95/1352; 7.03%) and EGJ outflow obstruction (31/1352; 2.3%).

The prevalence of dysphagia was significantly higher among patients with achalasia (80/95; 84.2%) than among those with normal manometry (282/770; 36.6%; p < 0.001). Referral for GERD symptoms occurred in 53.1% (52/98) of IEM patients versus 42.3% (326/770) of those with normal manometry (p = 0.056), indicating no significant difference.

PPI use vs no PPI use was associated with lower LES residual pressure (6.81 ± 8.11 mmHg vs 9.16 ± 10.41 mmHg) and LES basal pressure (24.33 ± 15.85 mmHg vs 28.75 ± 16.98 mmHg), both *p* < 0.001. Opioid use vs non-opioid use showed higher LES basal pressure (30.57 ± 18.67 vs 25.32 ± 16.11, *p* = 0.007), but no significant difference in LES residual pressure.

**Conclusions:**

Most HREM studies were normal, with achalasia and IEM being the most common abnormalities—achalasia mostly in dysphagia referrals and IEM in GERD referrals (not statistically significant). These findings highlight indication-specific likelihoods and HREM’s role in triage. Larger, multicenter studies with adjustments and linked pH/EGD data are needed to confirm their robustness and clinical relevance.

**Funding Agencies:**

None

